# Monocyte depletion increases local proliferation of macrophage subsets after skeletal muscle injury

**DOI:** 10.1186/1471-2474-14-359

**Published:** 2013-12-19

**Authors:** Claude H Côté, Patrice Bouchard, Nico van Rooijen, David Marsolais, Elise Duchesne

**Affiliations:** 1 Centre de Recherche du CHUL (CHUQ), 2705 Boulevard Laurier, RC-9800 Québec, Québec, Canada; 2 Département de Réadaptation, Faculté de Médecine, Université Laval, Québec, Québec, Canada; 3 Department of Molecular Cell Biology, Faculty of Medicine, Vrije Universiteit, Amsterdam, The Netherlands; 4 Département de Médecine, Faculté de Médecine, Université Laval, Québec, Québec, Canada; 5 Centre de Recherche de l'Institut Universitaire de Cardiologie et de Pneumologie de Québec, Québec, Canada; 6 Unité de Physiothérapie, Département des Sciences de la Santé, Université du Québec à Chicoutimi, Chicoutimi, Québec, Canada

**Keywords:** M1, M2, Bupivacaine, Clodronate, Flow cytometry, ED1, ED2, Ki67, Irradiation, Healing

## Abstract

**Background:**

Sequential accumulation of M1 and M2 macrophages is critical for skeletal muscle recovery after an acute injury. While M1 accumulation is believed to rely on monocyte infiltration, the mechanisms of M2 accumulation remain controversial, but could involve an infiltrating precursor. Yet, strong depletion of monocytes only partially impairs skeletal muscle healing, supporting the existence of alternative mechanisms to palliate the loss of infiltrating macrophage progenitors. The aims of this study are thus to investigate if proliferation occurs in macrophage subsets within injured skeletal muscles; and to determine if monocyte depletion leads to increased proliferation of macrophages after injury.

**Methods:**

Injury was induced by bupivacaine injection in the tibialis anterior muscle of rats. Blood monocytes were depleted by daily intravenous injections of liposome-encapsulated clodronate, starting 24 h prior to injury. In separate experiments, irradiation of hind limb was also performed to prevent resident cell proliferation. Upon euthanasia, blood and muscles were collected for flow cytometric analyses of macrophage/monocyte subsets.

**Results:**

Clodronate induced a 80%-90% depletion of monocyte but only led to 57% and 41% decrease of M1 and M2 macrophage accumulation, respectively, 2 d following injury. Conversely, the number of M1 macrophages in monocyte-depleted rats was 2.4-fold higher than in non-depleted rats 4 d after injury. This was associated with a 16-fold increase in the number of proliferative M1 macrophages, which was reduced by 46% in irradiated animals. Proliferation of M2 macrophages was increased tenfold by clodronate treatment 4 d post injury. The accumulation of M2 macrophages was partially impaired by irradiation, regardless of monocyte depletion.

**Conclusions:**

M1 and M2 subsets proliferate after skeletal muscle injury and their proliferation is enhanced under condition of monocyte depletion. Our study supports the conclusion that both infiltrating and resident precursors could contribute to M1 or M2 macrophage accumulation in muscle injury.

## Background

Macrophages are classically known for their pro-inflammatory roles in innate immunity and more recently for their active contribution to the resolution of inflammation and tissue repair [1-6]. This versatility is reflected by their ability to adopt distinct phenotypes depending on the microenvironment [7-9]. Macrophages can be divided in two main subsets according to their mode of activation and specific functions. In the context of skeletal muscle tissue injury, “classically activated” [10] M1 macrophages are found during the inflammatory phase and are associated with phagocytosis, while “alternatively activated” [10] M2 macrophages accumulate at the site of injury once necrotic tissue has been removed and participate to the repair and remodeling processes [7-9]. In rats, former nomenclature ED1^+^ and ED2^+^ refers to M1 (CD68^+^ CD163^-^) and M2 (CD68^+^ CD163^+^) macrophage subsets, respectively. For the sake of clarity we will thereafter refer to M1 and M2 macrophages. Macrophages insuring homeostasis of uninjured muscles are believed to be resident cells and would present a M2 phenotype [7,9]. Macrophage involvement is clearly a prerequisite for skeletal muscle repair and their deregulation can determine the outcome of skeletal muscle healing. The current dogma is that macrophages must freely accumulate in the injured muscle to ensure an adequate healing [1-3,11-13]. However, depletion of macrophages has only led to partial alteration of skeletal muscle recovery after injury [3], [1], supporting the existence of alternative mechanisms to ensure the functions of macrophages. Given the critical involvement of macrophage subsets in skeletal muscle healing, a better understanding of the mechanisms governing their accumulation may reveal new points of regulation for intervention.

The mechanisms of M1 and M2 macrophage accumulation after sterile injury remain elusive. In models like peritoneal infection, M1 or M2 macrophage accumulation results from the differentiation of distinct infiltrating M1 and M2 myeloid precursors [14]. In contrast, recent evidences support *in situ* proliferation of M1 and M2 macrophage subsets in T_H_2-mediated inflammation [14,15]. In the context of muscle injury, M1 macrophage accumulation is thought to result exclusively from the infiltration and differentiation of a precise monocyte subset into macrophages. On the other hand, the origin of the increased number of M2 macrophages is a matter of debate. A number of hypotheses are currently put forward including sequential mobilization of M1 and M2 macrophage circulating precursors [16], differentiation of monocyte-derived M1 macrophages into M2 macrophages following phagocytic activity after skeletal muscle injury [2] or M2 macrophage proliferation [15]. It appears that the mechanisms of M1 and M2 accumulation in this specific situation are complex, sometimes overlapping, and most likely determined by the context of the immune response.

The importance of sequential accumulation of M1 and M2 macrophages for optimal muscle healing is now well accepted. However, the cellular origin and respective contribution of proliferation vs. infiltration remain elusive following skeletal muscle injury. In addition, there is no information on how those mechanisms might be altered under anti-inflammatory conditions. The goal of this study was to determine if local proliferation could contribute to M1 and M2 macrophage accumulation following skeletal muscle injury, under normal or monocyte depletion conditions. Given that M1 macrophage accumulation in the context of sterile skeletal muscle injury is believed to rely exclusively on monocyte infiltration [2,16], and that M2 macrophage accumulation could be derived from M1 [2], our working hypothesis is that following sterile muscle injury in rat, blood monocyte depletion will impair M1 and M2 tissue macrophage accumulation.

## Methods

### Animals

Female Wistar rats weighing between 125 – 150 g were purchased from Charles River (St-Constant, QC, Canada) and housed 3 per cage. They were maintained on a 12 h – 12 h light – dark cycle. Water and food were provided *ad libitum*. All care, handling and experimental procedures were approved by the Université Laval Research Center Animal Care (Permit Number: 2012–025) and Use Committee according to the guidelines of the Canadian Council on Animal Care.

### Muscle injury

At 0 d, the right tibialis anterior (TA) muscle was chemically injured with bupivacaine. Rats received 0.05 mg/kg buprenorphine (Temgesic®, Reckitt Benckiser Healthcare (UK) Ltd) intraperitoneally (i.p.) as an analgesic 15 min before surgery and were then anesthetized with 1.5 – 2% isoflurane (Abbott Laboratories, Montreal, QC, Canada) under a flow of 400 – 800 mL/min of oxygen. Fur of the anterior side of the right hind limb was shaved off and skin was disinfected with isopropyl alcohol. Then, 120 μL of bupivacaine hydrocloride 0.5% (Marcaine; Hospira, Lake Forest, IL, USA) was injected through the skin in 3 sites of 40 μL along the right TA using a syringe with a 29 G needle. A single dose of 0.05 mg/kg of buprenorphine was also administrated i.p. 1 d after the injury.

### Monocyte depletion

Liposome-encapsulated dichloromethylene diphosphonate (Cl_2_MDP; clodronate) was used to deplete blood monocytes/macrophages [17]. Clodronate was a gift from Roche Diagnostics GmbH (Mannheim, Germany). Rats received 1 mL, at the concentration established by the provider, of the liposome-encapsulated clodronate suspension. Injections were made daily in the tail vein or in the internal jugular veins under anesthesia with isoflurane starting 24 h before injury until sacrifice at 1, 2, 3 or 4 d post-injury.

### Single leg irradiation

Liposome-encapsulated clodronate does not deplete resident macrophages in muscle. To study the contribution of resident macrophages, irradiation was performed since it prevents proliferation by inducing DNA damage, especially in immune cells [18,19]. 24 h before injury, rats were anesthetized by i.p. injection of a ketamine/xylazine cocktail (80 mg/kg ketamine and 10 mg/kg xylazine) and then installed under a 4 cm-thick lead shield plate with their right hind limb facing a hole allowing radiation to pass for local irradiation. The right leg received a single dose of 20 Gy of γ rays delivered at 1.1 Gy per minute using a Gammacell® 40 Exactor (Best Theratronics Ltd, Ottawa, ON, Canada). This dose prevents resident leukocyte replication without killing mature muscle cells [18,20-23].

### Isolation of peripheral blood mononuclear cells (PBMCs)

5 mL of blood was obtained by cardiac puncture under anesthesia with isoflurane and collected in BD Vacutainer® blood collection tubes containing EDTA (BD, Franklin Lakes, NJ, USA). PBMCs were isolated with a density-gradient centrifugation on Histopaque 1083 (Sigma-Aldrich, St. Louis, MO, USA). Blood was deposited on 3 mL of Histopaque 1083 in a 15 mL conical tube and centrifuged for 30 min at 400 × g at room temperature. The upper layer was discarded and the opaque interface containing mononuclear cells (buffy coat) was aspirated with a Pasteur pipette and transferred to a new 15 mL centrifuge tube. Cells were washed twice with PBS and contaminating erythrocytes were lysed with a 4 min incubation with 1 mL of sterile erythrocyte lysis buffer containing 155 mM NH_4_Cl, 10 mM KHCO_3_ and 0.342 mM EDTA. 10 mL of PBS was then added to stop the lysis. After washing twice with PBS, cells were resuspended in 0.5 mL PBS and counted with a hemocytometer using trypan blue exclusion.

### Preparation of single cell suspension from TA muscle

Rats were euthanized by cervical dislocation under anesthesia with isoflurane. TA muscles were dissected and rinsed in PBS. Minced muscles were incubated for 3 h at 37°C with 5 mL of Roswell Park Memorial Institute media 1640 (RPMI 1640) (HyClone, Logan, UT, USA) containing 3 mg/mL collagenase D (Roche Diagnostics, Laval, QC, Canada). Collagenase-digested samples were then homogenized by trituration using a 1000 μL micropipette. 10 mL PBS-EDTA (26 mM EDTA in PBS) was added to this suspension and centrifuged at 500 × g for 5 min at 4°C. After red blood cell lysis, the pellet was resuspended in 30% isotonic Percoll (GE healthcare, Waukesha, WI, USA) and centrifuged at 500 × g for 15 min at 4°C. The supernatant was discarded and the pellet resuspended in 1 mL PBS-EDTA. Cells were counted with a hemocytometer using trypan blue exclusion.

### Flow cytometric analyses

Sequential extracellular and intracellular stainings were performed as described earlier [24]. Single cell suspensions were washed in staining buffer (PBS containing 2% FBS). Cells were then resuspended in staining buffer containing Fc Block (anti-CD32) (BD Biosciences, Mississauga, ON, Canada). A cocktail of antibodies containing Pacific Blue-coupled anti-CD11b (Serotec, Kidlington, UK), biotin-coupled anti-CD3 (Serotec, Kidlington, UK), biotin-coupled anti-CD45RA (Biolegend, San Diego, CA, USA), Alexa Fluor® 647-coupled anti-CD163 (Serotec, Kidlington, UK) or Alexa Fluor® 647 coupled-IgG1 isotype control (Serotec, Kidlington, UK) was added to the cell suspension and incubated for 20 min on ice. Cells were washed in staining buffer and incubated with Alexa Fluor® 700-coupled streptavidin (Invitrogen Life Technologies, Burlington, Canada) for 20 min on ice. Cells were washed in staining buffer and fixed in PBS containing 2% paraformaldehyde for 20 min on ice, and then permeabilized in staining buffer containing 0.2% saponin for staining with anti RPE-coupled anti-CD68 (Serotec, Kidlington, UK) and Pe-Cy7-coupled anti-Ki-67 (BD Biosciences, Mississauga, ON, Canada) or RPE-coupled IgG1 isotype control (Serotec, Kidlington, UK) and Pe-Cy7-coupled k IgG1 isotype control (BD Biosciences, Mississauga, ON, Canada). Intracellular staining was performed for 30 min on ice. Single color controls were performed using CompBead Plus (BD Biosciences, Mississauga, ON, Canada). Data was acquired with a FACS Diva-driven FACS Aria II (Becton Dickinson) and analyzed with FlowJo (Tree Star Inc. Ashland, OR, USA). Isotype controls or omission of primary antibodies (when appropriate) were used to set gates. To determine numbers of specific cell subsets (from blood or skeletal muscles), the percentages obtained from flow cytometric analyses were multiplied by the absolute cell numbers obtained from the hemocytometer count.

### Statistical analysis

All values are expressed as means and standard error. The construct of these experiments allowed comparisons between groups to be performed by Student’s t-test or one-way ANOVA followed by Tukey-Kramer post-hoc test, when appropriate (InStat GraphPad Software Inc., La Jolla, CA, USA). Significance was defined as p < 0.05.

## Results

### Blood monocyte depletion modifies macrophage accumulation in injured muscle

**Figure 1:**
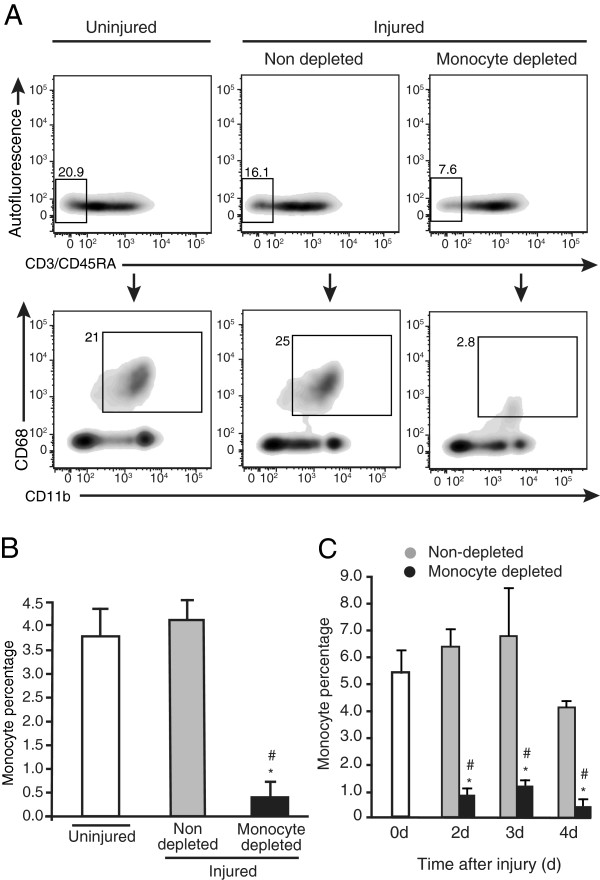
**Liposome-encapsulated clodronate eliminates more than 80% of blood monocytes. (A)** Rats received daily injections of liposome-encapsulated clodronate starting 24 h prior to intra-muscular injection of bupivacaine until euthanasia at 4 d post-injury. Clodronate treatment induces a decrease of the CD3^-^CD45RA^-^ non-lymphoid cell frequency (A, upper lane), and of the frequency of CD11b^+^CD68^+^ monocytes found in the CD3^-^CD45RA^-^ population (A, lower lane). Gates were set using either omission of primary antibodies (omission of CD3/CD45RA or CD11b) and using an isotype control for CD68. n = 3. **(B)** When expressed relatively to total PBMCs, the frequency of CD3^-^CD45RA^-^CD11b^+^CD68^+^ monocytes was decreased by 90% by the clodronate treatment in injured animals, in comparison to uninjured (*) and injured non-depleted (#) animals. Student’s t-test, p < 0.05. n = 3. **(C)** Using the same gating strategy, we shown that clodronate treatment significantly decreased the frequency of CD3^-^CD45RA^-^CD11b^+^CD68^+^ monocytes at 2 d and 3 d by 86% and 82%, respectively in injured animals, in comparison to uninjured (*) and injured non-depleted (#) animals. Student’s t-test, p < 0.05. n = 3.

Before assessing the contribution of blood monocyte infiltration to the accumulation of macrophages in injured muscle, we validated that daily liposome-encapsulated clodronate injection led to profound blood monocyte depletion. Blood monocytes correspond to the population negative for lymphocyte markers CD3 and CD45RA, but positive for myeloid marker CD11b and the monocyte/macrophage marker CD68 (Figure 1A). We used values obtained at 4 d post-injury to demonstrate that when compared with uninjured animals that contain 20.9% of non-lymphoid CD3^-^CD45RA^-^ circulating cells, muscle injury slightly decreased this frequency to 16.1%, while clodronate treatment lowered the frequency of CD3^-^CD45RA^-^ cells to 7.6% in injured rats. When gated from CD3^-^CD45RA^-^ cells, the frequency of CD11b^+^CD68^+^ monocytes corresponded to 21% in uninjured animals. This percentage was not significantly affected by the induction of the injury, but was decreased to 2.8% by clodronate injection in injured rats. Clodronate treatment thus led to a 90% decrease of the percentage of CD3^-^CD45RA^-^CD11b^+^CD68^+^ monocytes, relatively to the total PBMCs, in comparison with non-depleted animals injured with bupivacaine (Figure 1B). The degree of monocyte depletion was verified for each experience at all time points and similar depletion levels were observed: 86% at 2 d and 82% at 3 d (Figure 1C). Thus, the clodronate depletion procedure was effective throughout the experimental protocol.

**Figure 2:**
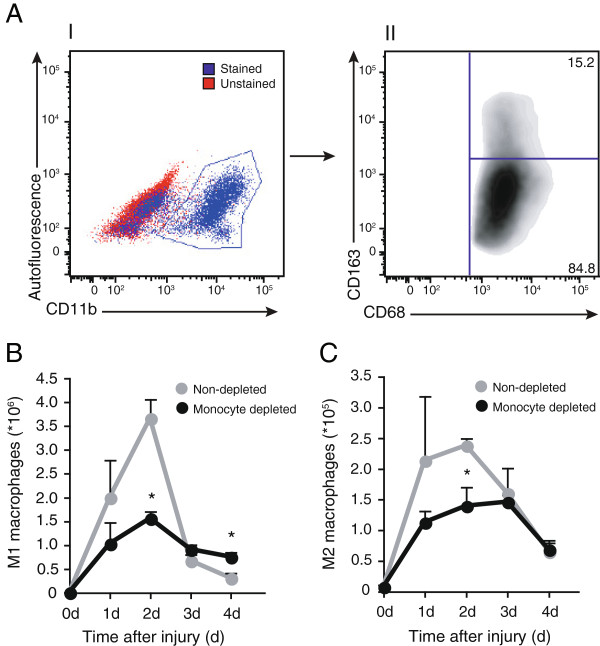
**Numbers of M1 and M2 macrophages in TA muscle are modified by blood monocyte depletion.** Rats were treated once a day with intravenous injection of liposome-encapsulated clodronate starting 24 h prior to injury until euthanasia at 1, 2, 3 and 4 d post-injury, for analysis of M1 and M2 macrophage accumulation into injured muscle. **(A)** Gating strategy (at 3 d post-injury) in single cell suspensions from skeletal muscles was based on omission of primary antibody for CD11b, and on isotype control stainings for CD68 and CD163 to identify M1 (CD163^-^) and M2 (CD163^+^) subsets. Liposome-encapsulated clodronate induced a significant decrease in accumulation of M1 **(B)** and M2 **(C)** macrophages at 2 d post-injury and a significant increase in M1 macrophage number at 4 d post-injury. *Significantly different from non-depleted groups, Student’s t-test, p < 0.05. n = at least 3, for all the time points and conditions evaluated.

We next characterized the effect of blood monocyte depletion on the accumulation of M1 and M2 macrophage subsets into TA muscle following muscle injury. The gating strategy consisted in prior selection of myeloid cells (CD11b^+^) from total cells (Figure 2A I), followed by selection of total CD68^+^ cells (not shown), and discrimination of the M1 and M2 populations based on the expression of CD163 by CD11b^+^CD68^+^ monocytes/macrophages (Figure 2A II). Prior to injury (0 d), only a few CD11b^+^CD68^+^CD163^-^ (M1) and CD11b^+^CD68^+^CD163^+^ (M2) macrophages were present in the TA muscle (Figure 2B and C). In injured animals non-depleted of monocytes, a classical accumulation of M1 macrophages occurred following injury, which peaked at 2 d with 3.67 × 10^6^ M1 macrophages per muscle and then dramatically decreased by 81% at 3 d before returning to baseline at 4 d post-injury (Figure 2B). A similar pattern was observed with M2 macrophages, with a peak at 2 d (2.38 × 10^5^ M2 macrophages per muscle). However M2 macrophage accumulation resolved more progressively than the M1 population, with a 33% decrease between 2 and 3 d, and above baseline accumulation remaining at 4 d (Figure 2C). When monocytes were depleted, we observed a 57% decrease in accumulation of M1 macrophages at 2 d post-injury, when compared to non-depleted injured animals (Figure 2B). Monocyte depletion had no impact on M1 macrophage number at 3 d, but it led to a 2.4-fold increase of M1 macrophage accumulation at 4 d, when compared with non-depleted injured rats (Figure 2B). As for the M2 macrophages, monocyte depletion induced a significant 41% decrease of their accumulation that was only detectable at 2 d after injury (Figure 2C). Taken together, these results show that a 90% blood monocyte depletion only leads to a partial (about 50%) decrease of macrophage accumulation at 2 d, and causes an increase in the number of the M1 subset at 4 d post-injury, suggesting an alternative mechanism of accumulation besides monocyte infiltration.

### Monocyte depletion is associated with proliferation of macrophages in injured muscles

**Figure 3:**
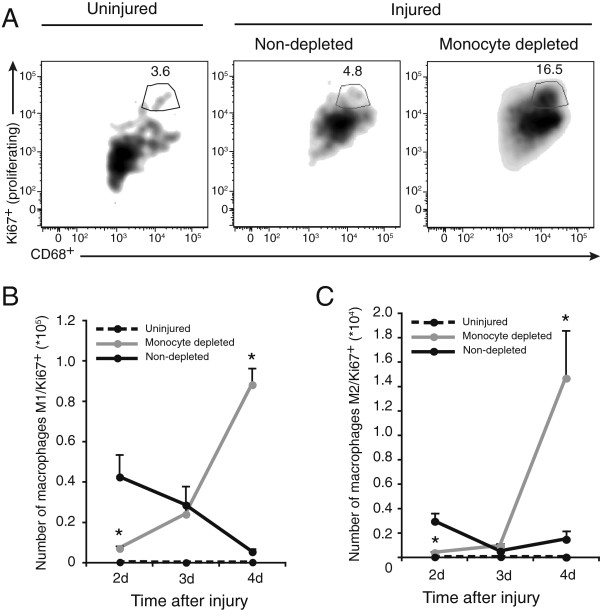
**Monocyte depletion induced significant increase in the number of proliferative macrophages in injured TA muscles.** Rats were treated once a day with intravenous injection of liposome-encapsulated clodronate starting 24 h prior to injury until euthanasia at 2, 3 and 4 d post-injury for analysis of the number of proliferating Ki-67^+^M1 and Ki-67^+^M2 macrophages into injured muscle. **(A)** Total CD11b^+^CD68^+^ proliferating macrophages also positive for Ki-67^+^ (not shown) were identified. Gates were set using isotype controls for Ki-67 and CD68. **(B)** Ki-67^+^M1 and **(C)** Ki-67^+^M2 macrophage subsets were quantified at 2, 3 and 4 d post-injury. Liposome-encapsulated clodronate induced a significant increase of Ki-67^+^M1 and Ki-67^+^M2 macrophage numbers at 4 d post-injury. *Significantly different from non-depleted groups, Student’s t-test, p < 0.05. n = at least 4, for all the time points and conditions evaluated.

Given that profound monocyte depletion led to modest decrease of macrophage numbers in injured muscles at 2 d and even to an increase at 4 d, we aimed at determining if local proliferation could occur in macrophages found within skeletal muscle. Using the gating strategy previously described (prior gating on CD11b^+^ cells, from viable cells), we then measured the proportion of M1 and M2 macrophages positive for Ki-67, which detects proliferative cells, irrespectively of their position within the cell cycle. In muscles of rats that were uninjured or that were injured but not submitted to monocyte depletion, 3.6% and 4.8% of all CD11b^+^CD68^+^ cells were positive for Ki-67 marker, respectively. The percentage of CD11b^+^CD68^+^Ki-67^+^ proliferative macrophages was increased by 3.4-fold when injured rats were treated with clodronate (Figure 3A). In uninjured rat muscles, the absolute numbers of Ki-67^+^M1 and Ki-67^+^M2 macrophages were barely detectable at all time points tested (Figure 3B and C). At 2 d, numbers of proliferating M1 and M2 macrophages were respectively 6- and 7.5-fold higher in non-depleted animals in comparison to depleted animals (Figure 3B and C), but these absolute numbers represented only a small fraction (approximately 1%) of the total M1 and M2 macrophages found in tissue at this time point under the same conditions (Figure 2B and C). Inversely, at 4 d, monocyte depletion led to significant 16- and 10-fold increases in the numbers of proliferating M1 and M2 macrophages (Figure 3B and C), respectively, which correspond to 10% of total M1 and 20% of M2 macrophage populations (Figure 2B and C). There is clearly an increased local proliferation of macrophages following skeletal muscle injury when monocyte infiltration is blocked.

**Figure 4:**
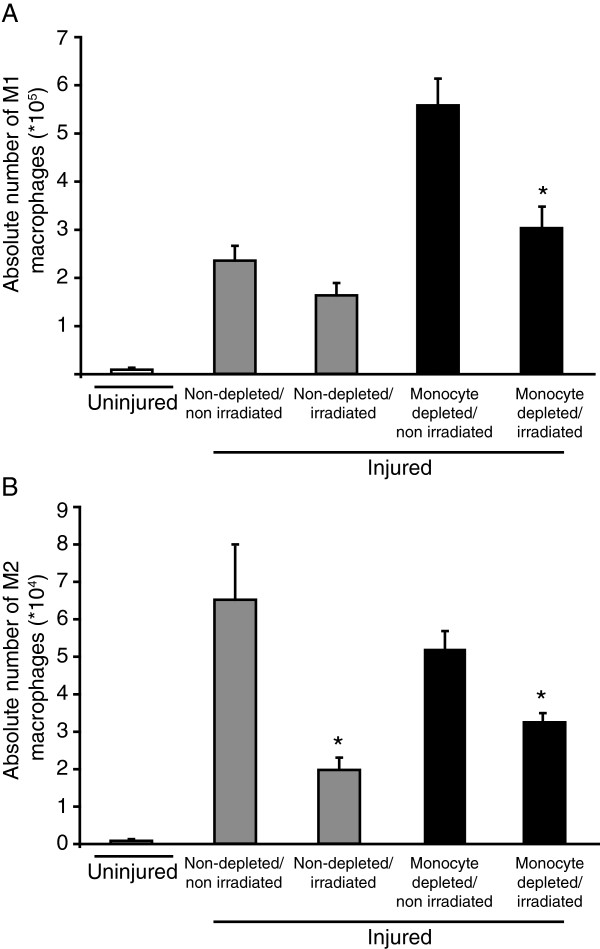
**Irradiation modulates M1 and M2 macrophage accumulation in TA muscle.** Hind limb was submitted to 20 Gy 24 h before injury to inhibit proliferation of resident cells. Rats were treated, or not, with daily intravenous injection of liposome-encapsulated clodronate starting 24 h prior to injury until euthanasia at 4 d post-injury, for analysis of the numbers of M1 and M2 macrophages into injured muscle. **(A)** Irradiation significantly decreased M1 macrophage accumulation when monocytes were depleted. **(B)** Irradiation impaired the accumulation of M2 macrophages following depletion or not. *Significantly different from the non-irradiated condition homologous, Student’s t-test, p < 0.05. n = at least 3, for all the time points and conditions evaluated.

To rule out the contribution of resident cells to M1 and M2 macrophage accumulation following injury, we used single leg irradiation before muscle injury. Irradiation did not affect the number of M1 macrophages in non-depleted animals 4 d after injury, supporting that infiltration is the main source of accumulation and that the population is contracting normally in those conditions (Figure 4A). However, irradiation reduced almost completely the depletion-induced increase of M1 macrophage number when compared to the non-depleted groups suggesting the implication of a local precursor (Figure 4A). Relatively to the uninjured group, M2 macrophages accumulated in spite of irradiation in non-depleted animals but to a lower extent than in non-irradiated/non-depleted animals, suggesting that their accumulation relies on both proliferation of local progenitor and an infiltrating precursor (Figure 4B). With irradiation, monocyte depletion did not induce a greater decrease in M2 macrophage accumulation, suggesting that M2 progenitors were unaffected when monocyte infiltration is artificially blocked (Figure 4B). Taken together, these results suggest that following muscle injury, M1 macrophage accumulation is mostly dependent on monocyte infiltration but that a local progenitor can contribute when monocyte infiltration is impaired. Conversely, M2 macrophage accumulation relies on both infiltrating and local precursors.

### The M2 Marker CD163 is not expressed by circulating monocytes

**Figure 5:**
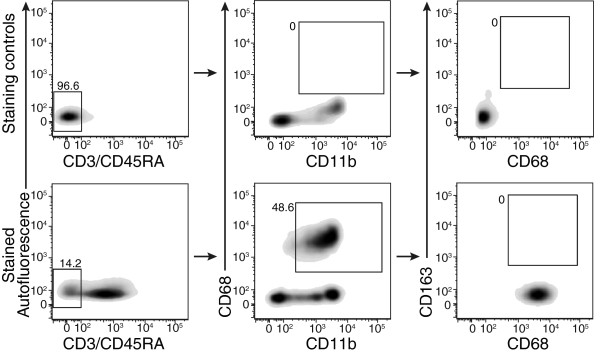
**CD68**^**+ **^**blood monocytes do not express CD163.** Blood was isolated from uninjured rat. Total white blood cells were collected and submitted to antibody staining for flow cytometric analyses to assess CD163 expression on CD3^-^CD45RA^-^CD11b^+^CD68^+^ cells. Gates were set using omission of primary antibody for CD3/CD45RA and for CD11b; and using isotype control staining for CD68 and CD163. Results shown are representative of 4 individual observations.

Since an infiltrating precursor seems to participate to M2 macrophage accumulation, we verified if a monocyte subset expressing CD163 marker was present in circulation. We used the previously defined strategy where CD3^-^/CD45RA^-^ stained PBMCs were analyzed based on expression of CD11b, CD68 and CD163. Gates were based on the omission of primary antibody for CD3/CD45RA and CD11b while isotype controls were used for CD68 and CD163 (Figure 5). We found that blood monocyte subset CD3^-^CD45RA^-^CD11b^+^CD68^+^ does not express CD163 (Figure 5). Our results are in accordance with previous literature showing that CD68 is largely used as human and rat monocyte marker, but CD163 expressed by human blood monocytes appears restricted to M2 macrophages in rat [25-27]. Taken together, our results support that in rat, circulating CD11b^+^CD68^+^ cells do not express the M2 CD163 marker.

## Discussion

Macrophages adopt various phenotypes based on their environment and participate to many physiological processes [4,8,28,29]. Substantial efforts have been deployed to characterize these various phenotypes with the hope that a better understanding of macrophage subsets could help delineate detrimental from the beneficial effects. It is now known that both M1 and M2 macrophages positively influence myogenesis, but can also contribute to pathological conditions [30-33]. Although mechanisms of macrophage replenishment were described in other tissues [34-36], the intrinsic mechanisms supporting their accumulation in the specific context of muscle injury are still a matter of debate and how these mechanisms are modulated under various anti-inflammatory strategies remains unknown. The main findings of this paper are that following muscle injury 1) both M1 and M2 macrophages can proliferate locally and 2) the major source of M1 macrophages are circulating precursors while M2 accumulation relies about equally on local (radiation sensitive) and infiltrating precursors. 3) We show for the first time that proliferation of macrophages is increased under condition of monocyte depletion.

We observed a low but quantifiable proliferation of M1 and M2 macrophage subsets following muscle injury at 2 d post-injury in non-depleted animals. This is in accordance with the reported proliferative ability of a number of macrophage subtypes including macrophages located in brain [35,37] or peritoneum [38], bone marrow-derived macrophages in culture [39], and the macrophage cell line RAW-264.7 [40]. Moreover, Hashimoto et al. have recently demonstrated that some tissue-resident macrophages repopulate locally via cellular proliferation with minimal contribution from monocytes, but these findings were observed under steady-state situation in alveolar macrophages, which are known to possess unique features such as a half-life 3 to 4 times longer than other macrophage subsets [34]. Ajami et al. also found no evidence of microglia progenitor recruitment from the circulation, but these results were observed in denervation and CNS neurodegenerative disease models [35]. Macrophage proliferation was also observed during chronic inflammation [41] and Khmelewski et al. (2004) revealed that both subsets of macrophages accumulated around collateral vessels in spite of monocyte depletion during femoral artery occlusion-induced arteriogenesis [42]. However, in these studies the authors failed to discriminate the proliferative capacity of M1 and M2 macrophages [41,42]. Jenkins et al. (2011) clearly showed that tissue macrophages with M2-like phenotype and M1 macrophages can undergo proliferation, but this was done in a model of T_H_-2-driven inflammation where the presence of IL-4 was essential [15]. Thus, macrophage proliferation has been demonstrated in various tissues and experimental models quite distinct from the one presented here. This is the first evidence that macrophage proliferation occurs specifically in skeletal muscle tissue and that proliferation contributes to macrophage accumulation following muscle injury. Our results are therefore unique and represent, to the best of our knowledge, the first demonstration that proliferation of M1 and M2 macrophages normally occurs following sterile muscle injury.

### M1 macrophage accumulation

Up to now, most studies pointed toward the infiltration of “inflammatory monocytes” to explain the accumulation of tissue macrophages after non-infectious tissue injury like skeletal muscle trauma. However, recent evidence shows that macrophages can undergo local proliferation in T_H_2-mediated inflammation [15]. This prompted us to verify the effect of sustained monocyte depletion, in an attempt to delineate if monocyte infiltration is the sole contributor to macrophage accumulation in muscle injury. In accordance with other studies [3,43,44], monocyte depletion induced a significant decrease in the absolute number of M1 macrophages present at 2 d post-injury. We surprisingly observed a higher number of M1 macrophages observed in the monocyte-depleted animals at 4 d post-injury, when compared to non-depleted injured animals. It was very unlikely that this phenomenon could be explained by an increased recruitment at 4 d, since current literature suggests that signals for monocyte recruitment are not produced at this time point [2]. Importantly, numerous reports showed delayed peaks of macrophage accumulation following acute muscle injury when using diverse anti-inflammatory strategies; our results are the first to suggest that the mechanisms leading to this delayed accumulation differ from that of normal accumulation and relies on local proliferation. One could argue that the preconditioning with clodronate has enriched the residual monocyte population for the highly proliferative monocytes. To verify it, we have assessed the effect of clodronate treatment on the percentage of proliferative monocytes into PBMCs population. We observed that clodronate treatment tended to enrich CD11b^+^CD68^+^Ki-67^+^ proliferative blood monocyte population by about 2.5-fold at all time points tested in comparison to untreated animals (data not shown). This increase represents a small absolute percentage of proliferative monocytes into PBMCs population (0.01% in non-treated vs. 0.025% in treated animals), and given the short timeframe of this study, those could have contributed partially the large increase of CD11b^+^CD68^+^Ki-67^+^ proliferative macrophages observed into muscle.

Our data strongly suggest that a precursor located within skeletal muscles prior to injury can contribute to M1 macrophage accumulation when monocyte infiltration is blocked. Irradiation, which prevents replication of resident macrophages, did not have any effect on the absolute number of M1 macrophages in non-depleted animals following injury, suggesting that infiltration of blood-derived monocytes was the main mechanism in those conditions. Inversely, irradiation short-circuited the increase of M1 macrophage number that was induced at 4 d with continuous depletion of monocytes. We conclude that when monocyte infiltration is artificially decreased or delayed, an alternative mechanism based on proliferation of a local M1 precursor is triggered to ensure the accumulation of that specific subset.

### M2 macrophage accumulation

Based on the present results, we conclude that M2 macrophage accumulation following injury relies on mixed mechanisms involving infiltration of blood monocytes as well as proliferation of local and radiation-sensitive precursors. Different mechanisms have been proposed to explain M2 macrophage accumulation; these include a specific circulating M2 precursor (CX_3_CR1^hi^/Ly6C^lo^ monocytes) [16], a switch of phenotype from M1 toward M2 [2], and lastly, the capacity of M2 macrophages to undergo local proliferation [15]. Data obtained in models of muscle injury led us to originally hypothesize that depletion of blood monocyte would eliminate M1 macrophages and thus indirectly decrease the number of M2 macrophages [2]. As predicted, CD163^+^ macrophage accumulation was significantly reduced at 2 d post-injury in monocyte-depleted animals, but this difference was lost at later time points. In an attempt to explain this phenomenon, we showed that irradiation had a significant impact on the absolute number of M2 macrophages in injured rats treated or not with clodronate. These results thus suggest that M2 macrophage accumulation is ensured, at least partially, by a local progenitor such as resident cells expressing M2 phenotype or resulting from the conversion of previously infiltrated M1 macrophages into M2 [7,16]. Conversely, M2 macrophage accumulation was still significant when muscle was irradiated, which argues against the hypothesis that local radiation-sensitive precursors are the only source for M2 macrophages. Overall, the data obtained by combining monocyte depletion and irradiation suggest that different pools of infiltrating and local M2 precursors could differently contribute to their accumulation. In order to identify a circulating progenitor for M2 macrophages, we assessed if a circulating monocyte subset expressed the M2 marker CD163 but were unable to detect such cells. Thus, the possibility of a CD68^+^CD163^-^ precursor or of an unknown circulating precursor for M2 macrophages remains open. Moreover, non-circulating progenitor cannot be excluded since it has been shown in brain that microglia are maintained throughout life independently of any blood input [45].

## Conclusions

In summary, the present study shows for the first time that macrophages have the capacity to proliferate following sterile muscle injury and that the number of proliferating macrophages is increased within muscles when monocyte infiltration is blocked. We conclude that under normal physiological conditions, the main source of M1 macrophages is circulating monocytes while M2 macrophage accumulation relies on both local and infiltrating precursors. As a whole, our study highlights overlapping mechanisms involved in macrophage accumulation after sterile skeletal muscle injury and suggests that these mechanisms are modulated when monocyte infiltration is impaired like is the case of anti-inflammatory conditions.

## Competing interests

The authors declare that they have no competing interests.

## Authors’ contribution

ED, CHC, DM conceived and designed the research. ED and PB performed experiments. ED analyzed the data. ED, CHC DM PB, NvR interpreted results of experiments. ED and PB drafted the manuscript. ED, CHC, DM, PB, NvR edited and revised the manuscript. NvR provided liposome-encapsulated clodronate. All authors approved the final version of manuscript.

## Pre-publication history

The pre-publication history for this paper can be accessed here:

http://www.biomedcentral.com/1471-2474/14/359/prepub

## References

[B1] Segawa M Fukada S Yamamoto Y Yahagi H Kanematsu M Sato M Ito T Uezumi A Hayashi S Miyagoe-Suzuki Y Suppression of macrophage functions impairs skeletal muscle regeneration with severe fibrosis Exp Cell Res 2008 14 17 3232 3244 10.1016/j.yexcr.2008.08.008 18775697

[B2] Arnold L Henry A Poron F Baba-Amer Y van Rooijen N Plonquet A Gherardi RK Chazaud B Inflammatory monocytes recruited after skeletal muscle injury switch into antiinflammatory macrophages to support myogenesis J Exp Med 2007 14 5 1057 1069 10.1084/jem.20070075 17485518 PMC2118577

[B3] Summan M Warren GL Mercer RR Chapman R Hulderman T Van Rooijen N Simeonova PP Macrophages and skeletal muscle regeneration: a clodronate-containing liposome depletion study Am J Physiol Regul Integr Comp Physiol 2006 14 6 R1488 R1495 10.1152/ajpregu.00465.2005 16424086

[B4] Chazaud B Brigitte M Yacoub-Youssef H Arnold L Gherardi R Sonnet C Lafuste P Chretien F Dual and beneficial roles of macrophages during skeletal muscle regeneration Exerc Sport Sci Rev 2009 14 1 18 22 10.1097/JES.0b013e318190ebdb 19098520

[B5] Tidball JG Villalta SA Regulatory interactions between muscle and the immune system during muscle regeneration Am J Physiol Regul Integr Comp Physiol 2010 14 5 R1173 R1187 10.1152/ajpregu.00735.2009 20219869 PMC2867520

[B6] Villalta SA Rinaldi C Deng B Liu G Fedor B Tidball JG Interleukin-10 reduces the pathology of mdx muscular dystrophy by deactivating M1 macrophages and modulating macrophage phenotype Human molecular genetics 2011 14 4 790 805 10.1093/hmg/ddq523 21118895 PMC3024048

[B7] Murray PJ Wynn TA Protective and pathogenic functions of macrophage subsets Nat Rev Immunol 2011 14 11 723 737 10.1038/nri3073 21997792 PMC3422549

[B8] Lawrence T Natoli G Transcriptional regulation of macrophage polarization: enabling diversity with identity Nat Rev Immunol 2011 14 11 750 761 10.1038/nri3088 22025054

[B9] Gordon S Taylor PR Monocyte and macrophage heterogeneity Nat Rev Immunol 2005 14 12 953 964 10.1038/nri1733 16322748

[B10] Mosser DM Edwards JP Exploring the full spectrum of macrophage activation Nat Rev Immunol 2008 14 12 958 969 10.1038/nri2448 19029990 PMC2724991

[B11] Martinez CO McHale MJ Wells JT Ochoa O Michalek JE McManus LM Shireman PK Regulation of skeletal muscle regeneration by CCR2-activating chemokines is directly related to macrophage recruitment Am J Physiol Regul Integr Comp Physiol 2010 14 3 R832 R842 10.1152/ajpregu.00797.2009 20631294 PMC2944434

[B12] Sun D Martinez CO Ochoa O Ruiz-Willhite L Bonilla JR Centonze VE Waite LL Michalek JE McManus LM Shireman PK Bone marrow-derived cell regulation of skeletal muscle regeneration FASEB J 2009 14 2 382 395 18827026 10.1096/fj.07-095901 PMC2630778

[B13] Tidball JG Wehling-Henricks M Macrophages promote muscle membrane repair and muscle fibre growth and regeneration during modified muscle loading in mice in vivo The Journal of physiology 2007 14 Pt 1 327 336 17038433 10.1113/jphysiol.2006.118265 PMC2075127

[B14] Geissmann F Auffray C Palframan R Wirrig C Ciocca A Campisi L Narni-Mancinelli E Lauvau G Blood monocytes: distinct subsets, how they relate to dendritic cells, and their possible roles in the regulation of T-cell responses Immunology and cell biology 2008 14 5 398 408 10.1038/icb.2008.19 18392044

[B15] Jenkins SJ Ruckerl D Cook PC Jones LH Finkelman FD van Rooijen N MacDonald AS Allen JE Local macrophage proliferation, rather than recruitment from the blood, is a signature of TH2 inflammation Science 2011 14 6035 1284 1288 10.1126/science.1204351 21566158 PMC3128495

[B16] Nahrendorf M Swirski FK Aikawa E Stangenberg L Wurdinger T Figueiredo JL Libby P Weissleder R Pittet MJ The healing myocardium sequentially mobilizes two monocyte subsets with divergent and complementary functions J Exp Med 2007 14 12 3037 3047 10.1084/jem.20070885 18025128 PMC2118517

[B17] van Rooijen N van Kesteren-Hendrikx E In vivo" depletion of macrophages by liposome-mediated "suicide Methods in enzymology 2003 14 3 16 14714393 10.1016/s0076-6879(03)73001-8

[B18] Hodgetts SI Grounds MD Irradiation of dystrophic host tissue prior to myoblast transfer therapy enhances initial (but not long-term) survival of donor myoblasts J Cell Sci 2003 14 Pt 20 4131 4146 12972504 10.1242/jcs.00721

[B19] Denekamp J Rojas A Cell kinetics and radiation pathology Experientia 1989 14 1 33 41 10.1007/BF01990450 2643525

[B20] Robertson TA Grounds MD Papadimitriou JM Elucidation of aspects of murine skeletal muscle regeneration using local and whole body irradiation J Anat 1992 14 Pt 2 265 276 1295865 PMC1259722

[B21] Gross JG Morgan JE Muscle precursor cells injected into irradiated mdx mouse muscle persist after serial injury Muscle Nerve 1999 14 2 174 185 10.1002/(SICI)1097-4598(199902)22:2<174::AID-MUS5>3.0.CO;2-S 10024130

[B22] Gulati AK The effect of X-irradiation on skeletal muscle regeneration in the adult rat J Neurol Sci 1987 14 1 111 120 10.1016/0022-510X(87)90083-9 3572447

[B23] Gross JG Bou-Gharios G Morgan JE Potentiation of myoblast transplantation by host muscle irradiation is dependent on the rate of radiation delivery Cell Tissue Res 1999 14 2 371 375 10.1007/s004419900062 10571126

[B24] Marsolais D Hahm B Walsh KB Edelmann KH McGavern D Hatta Y Kawaoka Y Rosen H Oldstone MB A critical role for the sphingosine analog AAL-R in dampening the cytokine response during influenza virus infection Proc Natl Acad Sci USA 2009 14 5 1560 1565 10.1073/pnas.0812689106 19164548 PMC2635800

[B25] Kim WK Alvarez X Fisher J Bronfin B Westmoreland S McLaurin J Williams K CD163 identifies perivascular macrophages in normal and viral encephalitic brains and potential precursors to perivascular macrophages in blood Am J Pathol 2006 14 3 822 834 10.2353/ajpath.2006.050215 16507898 PMC1606539

[B26] Tippett E Cheng WJ Westhorpe C Cameron PU Brew BJ Lewin SR Jaworowski A Crowe SM Differential expression of CD163 on monocyte subsets in healthy and HIV-1 infected individuals PloS one 2011 14 5 e19968 10.1371/journal.pone.0019968 21625498 PMC3098854

[B27] Polfliet MM Fabriek BO Daniels WP Dijkstra CD van den Berg TK The rat macrophage scavenger receptor CD163: expression, regulation and role in inflammatory mediator production Immunobiology 2006 14 6–8 419 425 16920481 10.1016/j.imbio.2006.05.015

[B28] Mills CD Kincaid K Alt JM Heilman MJ Hill AM M-1/M-2 macrophages and the Th1/Th2 paradigm J Immunol 2000 14 12 6166 6173 10843666 10.4049/jimmunol.164.12.6166

[B29] Mantovani A Sica A Sozzani S Allavena P Vecchi A Locati M The chemokine system in diverse forms of macrophage activation and polarization Trends Immunol 2004 14 12 677 686 10.1016/j.it.2004.09.015 15530839

[B30] Moyer AL Wagner KR Regeneration versus fibrosis in skeletal muscle Current opinion in rheumatology 2011 14 6 568 573 10.1097/BOR.0b013e32834bac92 21934499

[B31] Bot A Smith KA von Herrath M Molecular and cellular control of T1/T2 immunity at the interface between antimicrobial defense and immune pathology DNA and cell biology 2004 14 6 341 350 10.1089/104454904323145227 15231067

[B32] Khallou-Laschet J Varthaman A Fornasa G Compain C Gaston AT Clement M Dussiot M Levillain O Graff-Dubois S Nicoletti A Macrophage plasticity in experimental atherosclerosis PloS one 2010 14 1 e8852 10.1371/journal.pone.0008852 20111605 PMC2810335

[B33] Mikita J Dubourdieu-Cassagno N Deloire MS Vekris A Biran M Raffard G Brochet B Canron MH Franconi JM Boiziau C Altered M1/M2 activation patterns of monocytes in severe relapsing experimental rat model of multiple sclerosis. Amelioration of clinical status by M2 activated monocyte administration Mult Scler 2011 14 1 2 15 10.1177/1352458510379243 20813772

[B34] Hashimoto D Chow A Noizat C Teo P Beasley MB Leboeuf M Becker CD See P Price J Lucas D Tissue-resident macrophages self-maintain locally throughout adult life with minimal contribution from circulating monocytes Immunity 2013 14 4 792 804 10.1016/j.immuni.2013.04.004 23601688 PMC3853406

[B35] Ajami B Bennett JL Krieger C Tetzlaff W Rossi FM Local self-renewal can sustain CNS microglia maintenance and function throughout adult life Nature neuroscience 2007 14 12 1538 1543 10.1038/nn2014 18026097

[B36] Schulz C Gomez Perdiguero E Chorro L Szabo-Rogers H Cagnard N Kierdorf K Prinz M Wu B Jacobsen SE Pollard JW A lineage of myeloid cells independent of Myb and hematopoietic stem cells Science 2012 14 6077 86 90 10.1126/science.1219179 22442384

[B37] Dobbertin A Schmid P Gelman M Glowinski J Mallat M Neurons promote macrophage proliferation by producing transforming growth factor-beta2 The Journal of neuroscience: the official journal of the Society for Neuroscience 1997 14 14 5305 5315 9204915 10.1523/JNEUROSCI.17-14-05305.1997 PMC6793830

[B38] Senokuchi T Matsumura T Sakai M Yano M Taguchi T Matsuo T Sonoda K Kukidome D Imoto K Nishikawa T Statins suppress oxidized low density lipoprotein-induced macrophage proliferation by inactivation of the small G protein-p38 MAPK pathway J Biol Chem 2005 14 8 6627 6633 10.1074/jbc.M412531200 15611087

[B39] Celada A Borras FE Soler C Lloberas J Klemsz M van Beveren C McKercher S Maki RA The transcription factor PU.1 is involved in macrophage proliferation J Exp Med 1996 14 1 61 69 10.1084/jem.184.1.61 8691150 PMC2192661

[B40] Moeslinger T Spieckermann PG Urea-induced inducible nitric oxide synthase inhibition and macrophage proliferation Kidney international Supplement 2001 14 S2 S8 11168974 10.1046/j.1523-1755.2001.59780002.x

[B41] Spector WG Wynne KM Proliferation of macrophages in inflammation Agents and actions 1976 14 1–3 123 126 941788 10.1007/BF01972195

[B42] Khmelewski E Becker A Meinertz T Ito WD Tissue resident cells play a dominant role in arteriogenesis and concomitant macrophage accumulation Circulation research 2004 14 6 E56 E64 10.1161/01.RES.0000143013.04985.E7 15331452

[B43] Bryer SC Fantuzzi G Van Rooijen N Koh TJ Urokinase-type plasminogen activator plays essential roles in macrophage chemotaxis and skeletal muscle regeneration J Immunol 2008 14 2 1179 1188 18178858 10.4049/jimmunol.180.2.1179

[B44] DiPasquale DM Cheng M Billich W Huang SA van Rooijen N Hornberger TA Koh TJ Urokinase-type plasminogen activator and macrophages are required for skeletal muscle hypertrophy in mice Am J Physiol Cell Physiol 2007 14 4 C1278 C1285 10.1152/ajpcell.00201.2007 17652428

[B45] Ginhoux F Greter M Leboeuf M Nandi S See P Gokhan S Mehler MF Conway SJ Ng LG Stanley ER Fate mapping analysis reveals that adult microglia derive from primitive macrophages Science 2010 14 6005 841 845 10.1126/science.1194637 20966214 PMC3719181

